# Omissions and Byproducts across Moral Domains

**DOI:** 10.1371/journal.pone.0046963

**Published:** 2012-10-11

**Authors:** Peter DeScioli, Kelly Asao, Robert Kurzban

**Affiliations:** 1 Department of Psychology, Brandeis University, Waltham, Massachusetts, United States of America; 2 Department of Economics, Brandeis University, Waltham, Massachusetts, United States of America; 3 Department of Psychology, University of Pennsylvania, Philadelphia, Pennsylvania, United States of America; Boston College, United States of America

## Abstract

Research indicates that moral violations are judged less wrong when the violation results from omission as opposed to commission, and when the violation is a byproduct as opposed to a means to an end. Previous work examined these effects mainly for violent offenses such as killing. Here we investigate the generality of these effects across a range of moral violations including sexuality, food, property, and group loyalty. In Experiment 1, we observed omission effects in wrongness ratings for all of the twelve offenses investigated. In Experiments 2 and 3, we observed byproduct effects in wrongness ratings for seven and eight offenses (out of twelve), respectively, and we observed byproduct effects in forced-choice responses for all twelve offenses. Our results address an ongoing debate about whether different cognitive systems compute moral wrongness for different types of behaviors (surrounding violence, sexuality, food, etc.), or, alternatively, a common cognitive architecture computes wrongness for a variety of behaviors.

## Introduction

The moral status of omissions and byproducts has been discussed and debated throughout history. For example, the medieval philosopher Aquinas asserted that “transgression is a graver sin than omission” and that “moral acts take their species according to what is intended, and not according to what is beside the intention” [1]. Consistent with Aquinas's claims, research on moral judgments has shown an omission effect, in which violations by omission are condemned less harshly than violations by commission [2]–[7], and a byproduct effect, in which violations occurring as a byproduct are condemned less harshly than violations occurring as a means to an end [8]–[11].

Previous research on omissions and byproducts has tended to focus on violent moral offenses such as killing a victim in order to save other people. However, some researchers have emphasized that morality encompasses a diverse set of content domains. Prohibitions against violence and harm constitute one moral domain, but morality also includes rules about other domains such as purity, authority, divinity, and loyalty [12]–[14]. The diversity of moral phenomena raises questions about the generality of omission and byproduct effects. Several previous studies suggest that omission and byproduct effects might occur widely across moral domains. For example, lying by omission is judged as less wrong than lying by commission [7], [15], [16] (for similar findings in children, see [17]). However, no previous study has specifically examined omissions and byproducts across a diversity of moral offenses, including major moral categories such as sexuality, property, loyalty, and food taboos.

### The Function(s) of Moral Computation

The functional organization of moral cognition remains a subject of active discussion and debate. One basic question is whether moral cognition is best understood as a single cognitive system operating on many types of violations [18], [19] or a set of multiple systems, each specialized for a particular type of violation [12], [13], [20], [21]. Haidt [12], for example, has suggested that moral cognition is composed of “five psychological foundations, each with a separate evolutionary origin” (p. 1001) which underlie the distinct moral domains of harm, fairness, ingroup loyalty, authority, and purity. Pluralist approaches generally emphasize the diversity of moral inputs, including food choices, sexuality, disobedience, dishonesty, and violence, whereas unitary approaches emphasize consistency across domains in moral outputs, especially the suite of complex behaviors associated with moral condemnation [18].

Importantly, the debate is not whether different behaviors such as theft, assault, and adultery are processed by different domain-specific mechanisms—both models are consistent with this idea. Rather, the debate is whether distinctively *moral* judgments—evaluations of moral wrongness—are computed independently by different domain-specific mechanisms or are computed for these different behaviors by the same moral analyzer. For example, people could have domain-specific mechanisms for processing property disputes [22] and separate mechanisms for processing sexual infidelity [23], yet the moral wrongness of theft and infidelity could still be computed by a single moral analyzer specialized for evaluating wrongness for a variety of behaviors.

The structure of moral cognition can be clarified by examining the representations and mental processes underlying moral thought. If different information-processing patterns occur for different types of moral violations, then this provides evidence for violation-specific components of moral cognition. If there are processing patterns that occur across different types of violations, then this provides evidence for a common cognitive architecture that is applied across moral domains. Omission and byproduct effects can provide a useful testbed for these models. By investigating omissions and byproducts across a variety of moral domains, we can determine which violations show similar and dissimilar information-processing signatures.

The observed pattern of effects will inform theories about the functions of moral cognition. A computational architecture that varies across different types of violations requires identifying a set of violation-specific adaptive problems that account for this variation, i.e., different functional theories for prohibitions surrounding violence, property, sex, food, communication, etc. In contrast, a common moral architecture requires theories that explain why moral cognition operates so broadly, presumably in terms of adaptive problems that cut across different types of violations.

### The Present Experiments

The present experiments extend previous research by investigating moral judgment of omissions and byproducts across a wide range of moral violations. Experiment 1 examines the omission effect and Experiments 2 and 3 examine the byproduct effect. Our main research question is whether omission and byproduct effects are confined to violent offenses or whether they are observed more broadly across moral domains. This question is distinct from a further question about variation across domains in the size of these effects, such as previous research comparing the effect of intentions in harm and purity domains [24]. The present experiments are designed to test these effects across a range of different offenses rather than to compare magnitudes across domains (see General Discussion).

## Experiment 1

### Method

#### Design

Experiment 1 investigated omissions across moral domains using controlled pairs of violation scenarios (Methods S1). We designed scenarios in which an actor's behavior, commission or omission, violated a moral rule. Our scenarios did not involve moral dilemmas (with ambiguity about which decision is morally best) but simply depicted violating behavior and asked participants to judge the magnitude of the moral offense. The scenarios described observable behavior and events, and they contained no assertions of unobservable mental states (e.g., the actor “sees” rather than “knows”). With this approach, our aim was to keep the stimuli as close as possible to the information inputs that people could receive directly from experience. Finally, both commission and omission scenarios were written such that the actors' violations were foreseen and chosen (given that an omission could result from involuntary inattention or misunderstanding). For example, in the group sex scenario an actor is having sex with his wife, another couple joins in, and he fails to leave the situation, thereby participating in group sex by omission. In this case, the ongoing participation of the actor shows that the continuing violation was foreseen and voluntarily chosen.

In a within-subject design, participants read 24 scenarios involving moral infractions – 12 commission-omission pairs for each offense type. We selected the offense types to include a variety of moral domains. The violations were theft of $20, cheating on an exam, selling sex, buying sex, viewing child pornography, group sex, littering in a park, euthanasia, cannibalism, marijuana use, flag-burning, and injuring someone (the victim falls from a ladder). For example, in the buying sex scenarios, Alex went to a massage parlor and paid the masseuse an extra $20 for oral sex (a violation by commission); Aaron gave the masseuse a $20 tip, she began performing oral sex, and he did not stop her (a violation by omission).

The order in which participants responded to the commission and omission scenarios was counterbalanced across subjects. For each scenario, participants answered questions about one focal individual. Participants rated moral wrongness and they indicated how much punishment the focal individual deserved.

#### Participants

Participants were 151 undergraduates (103 female) enrolled in introductory psychology at the University of Pennsylvania. The mean (*SD*) age of the sample was 19.35 (1.98) years.

#### Materials

The experiment was conducted by computer via a web-based service designed to collect survey data (www.surveymonkey.com). Each scenario was presented on a different page along with wrongness and punishment items.

For the moral wrongness scale, participants answered the question “How morally wrong is the action?” by rating wrongness on a scale from 0 (*not wrong at all*) to 100 (*most wrong*). We provided participants with six additional scale anchors taken from the National Survey of Crime Severity [25]: noise disturbance (“1”), assault with lead pipe causing injury (“10”), knife stabbing causing injury (“20”), knife stabbing causing death (“35”), rape resulting in death (“50”), and planting a bomb causing 20 deaths (“70”).

For the deserved punishment measure, participants answered “What punishment does this action deserve?” by assigning prison time (in months and days). We provided participants with six anchors taken from the 2006 U.S. Guidelines for Prison Sentencing: trespassing (0–6 months), firearm possession (10–16 months), robbery (33–41 months), sexual abuse (97–121 months), rape (151–188 months), and espionage (360 months – life).

**Procedure**: Sessions were held in the Penn Laboratory for Experimental Evolutionary Psychology (PLEEP) at the University of Pennsylvania. Participants read an informed consent document, read the experimental instructions, and began the experiment. After finishing the experiment, participants provided demographic information and then they were debriefed and dismissed. The procedure took 30 minutes. Participants received a consent form which they were required to read. Written consent was not required because the data were analyzed anonymously. Procedures were approved by the University of Pennsylvania Institutional Review Board.

#### Data Analysis

We tested for the presence of omission effects for each type of violation using within-subject *t*-tests. For each offense, we excluded from analysis individuals who assigned a wrongness rating of zero to both commission and omission stimuli because these ratings indicated that the participant did not view that particular behavior (e.g., littering) as morally relevant. Because we used a range of moral violations, we expected variation in whether people saw each violation as condemnable. The specific research question was not about this variation in applying moral judgment, but rather, *conditional on viewing a behavior as morally wrong*, whether people judge omissions less harshly than commissions. Table 1 shows the number of participants in the analysis for each violation.

**Table 1:** Experiment 1 wrongness judgments.

		Commission		Omission				
Violation	*n* a	Mean	*SD*	Mean	*SD*	Difference	*t*	*p*
Buy Sex	143	13.43	16.79	7.73	13.25	5.70	7.81	<.001
Cannibalism	139	18.01	18.82	11.24	16.33	6.77	7.60	<.001
Cheating	150	9.77	12.65	6.16	7.58	3.61	6.56	<.001
Child Porn	150	16.62	18.83	13.95	18.09	2.67	3.34	.001
Euthanasia	125	17.54	21.97	12.55	17.27	4.99	3.38	.001
Flag-burning	135	11.60	14.29	6.90	8.57	4.70	5.31	<.001
Group Sex	147	6.99	9.94	4.44	6.28	2.55	4.83	<.001
Injury	151	19.96	16.36	11.81	12.19	8.15	9.60	<.001
Littering	105	12.06	16.52	9.80	15.12	2.26	3.77	<.001
Marijuana	106	4.89	9.38	3.30	3.71	1.58	1.69	.093
Sell Sex	147	9.94	12.50	5.40	9.42	4.54	7.00	<.001
Theft	151	9.95	11.25	5.03	8.89	4.91	7.42	<.001

*Note*. All *t* tests were within-subject and two-tailed. Participants viewing neither commission nor omission as wrong were excluded.

a Subset of total sample (*N* = 151) after excluding individuals viewing neither as wrong.

Previous research has found that the omission effect is enhanced when omissions are judged before commissions and diminished in the opposite order [7], [26]. Because this pattern is not the focus of the present experiment, we collapsed across presentation order for the main analysis, but we also summarize the nature of these effects (complete analyses available upon request).

### Results

Table 1 shows mean (*SD*) wrongness judgments for commission and omission conditions. Wrongness ratings ranged from *M* = 3.30 for marijuana use by omission to *M* = 19.96 for physical injury by commission, indicating that participants generally rated offense wrongness as somewhere between a noise disturbance (“1” on the scale), assault with a lead pipe (“10”), and knife stabbing causing injury (“20”). We conducted a paired *t*-test for each violation type. For eleven of the twelve violations, omissions were judged as less wrong than commissions. For marijuana use, the difference was marginally significant.

Table 2 shows mean (*SD*) punishment judgments by condition. Prison time assignments ranged from *M* = 0.01 months for cheating on an exam by omission to *M* = 15.03 months for euthanasia by commission. Paired *t*-tests showed that omissions were assigned significantly less prison time than commissions for eleven of twelve offenses. For littering, the difference was not significant.

**Table 2:** Experiment 1 punishment judgments (months in prison).

		Commission		Omission				
Violation	*n* a	Mean	*SD*	Mean	*SD*	Difference	*t*	*p*
Buy Sex	143	3.30	12.33	0.17	0.78	3.14	3.06	.003
Cannibalism	139	7.09	31.79	2.62	15.21	4.47	1.96	.052
Cheating	150	0.13	0.56	0.01	0.06	0.11	2.63	.010
Child Porn	150	6.31	17.54	2.86	8.42	3.45	3.13	.002
Euthanasia	125	15.03	49.90	10.03	36.25	5.01	2.36	.020
Flag-burning	135	1.91	6.93	0.55	1.94	1.36	2.34	.020
Group Sex	147	0.43	1.79	0.06	0.29	0.37	2.78	.006
Injury	151	7.28	14.95	1.19	4.73	6.09	5.41	<.001
Littering	105	2.05	13.41	0.17	1.20	1.89	1.53	.13
Marijuana	106	0.42	2.45	0.18	1.47	0.25	2.27	.026
Sell Sex	147	1.44	4.98	0.02	0.11	1.41	3.44	<.001
Theft	151	2.18	6.62	0.26	2.69	1.92	3.28	.001

*Note*. All *t* tests are within-subject and *p* values are two-tailed. Participants viewing neither commission nor omission as wrong were excluded.

a Subset of total sample (*N* = 151) after excluding individuals viewing neither as wrong.

#### Additional Analyses

Consistent with previous research, we observed order effects in wrongness judgments such that the omission effect was enhanced when omissions were judged prior to commissions and diminished in the opposite order. All twelve offenses showed a significant omission effect for wrongness judgments when omissions were judged first, whereas four offenses (child porn, euthanasia, littering, marijuana) showed no effect when commissions were judged first. In no case did participants judge omissions as worse than commissions.

The present experiments were not well-designed to compare effect magnitudes across moral domains (see General Discussion). Nonetheless, we conducted exploratory analyses to examine omission effects in sex-related offenses (buy sex, child porn, group sex, sell sex) compared to the other offenses. We conducted a 2 (omission or commission)×2 (sex-related or not) repeated ANOVA for wrongness judgments and we found an omission by sex-related interaction, *F*(1, 1647) = 3.81, *p* = .05, such that the commission-omission difference was smaller for sex-related offenses (*M* = 3.84) than for other offenses (*M* = 4.82). Paired *t*-tests showed that in aggregate the commission-omission difference was significant for sex-related offenses, *t*(586) = 11.13, *p*<.001, and other offenses, *t*(1061) = 15.14, *p*<.001. For punishment judgments, we did not observe this interaction, *F*(1, 1647) = 0.83, *p* = .36. Paired *t*-tests showed that in aggregate the commission-omission difference was significant for sex-related offenses, *t*(586) = 5.32, *p*<.001, and other offenses, *t*(1061) = 5.95, *p*<.001.

Last, we explored whether the participant's sex influenced omission effects by using a 2 (sex)×2 (omission or commission) repeated ANOVA. We found a sex by omission interaction, *F*(1, 1647) = 4.41, *p* = .036, such that across violation types the commission-omission difference was smaller for males (*M* = 3.73) than for females (*M* = 4.81). We did not observe a sex by omission interaction for punishment decisions, *F*(1, 1647) = 0.85, *p* = .36.

### Discussion

The results of Experiment 1 indicate that the omission effect is not specific to violent offenses but occurs across a range of moral violations.

## Experiment 2

Mikhail [10] provides a particularly clear example of the means-byproduct distinction with variants of the trolley problem. The scenario involved a bystander who can divert a train heading for five people onto a sidetrack that loops back to the main track. Diverting the track will save the people, but only because there is a heavy weight on the track that will slow the train. In the means condition, the heavy weight is a man. In the byproduct condition, the heavy weight is an object on the track, but there is a man standing in front of it who will be killed. Participants judged killing as a means as impermissible 52% of the time, whereas killing as a byproduct was judged impermissible significantly less, 38% of the time.

The means-byproduct distinction is subtle, which is one reason that there is considerable interest surrounding why people's judgments are sensitive to these differences. Because the byproducts are foreseeable, they can easily be viewed as part of the overall plan of the actor, and therefore as part of the “means” they used to accomplish their goals. In the trolley case, for instance, killing the man in front of the weight could be viewed as part of the plan to use the object to slow the train, and hence as part of the “means.” In other words, it is always possible to disregard the difference between a means and a foreseeable byproduct. What one person describes as “collateral damage,” someone else can always reframe as “instrumental damage.” Despite this, the difference matters for moral judgments of killing.

**Figure 1:**
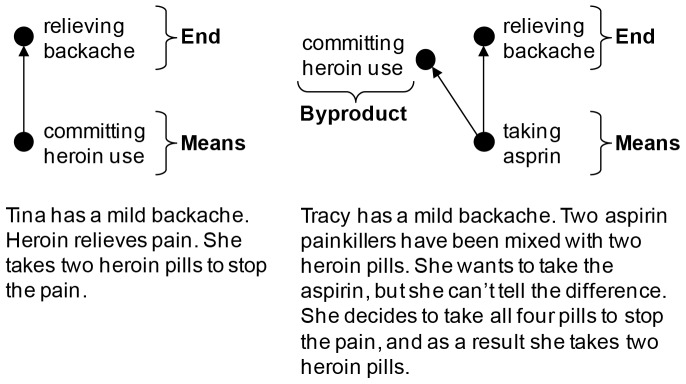
Violation as a means versus violation as a byproduct for heroin use. The byproduct effect can be understood in terms of the structural descriptions that encode means and byproducts in moral representations (Mikhail, 2007). The paired scenarios about Tina and Tracy are designed to isolate the means-byproduct distinction from the many other variables that are encoded in moral representations.

**Figure 2:**
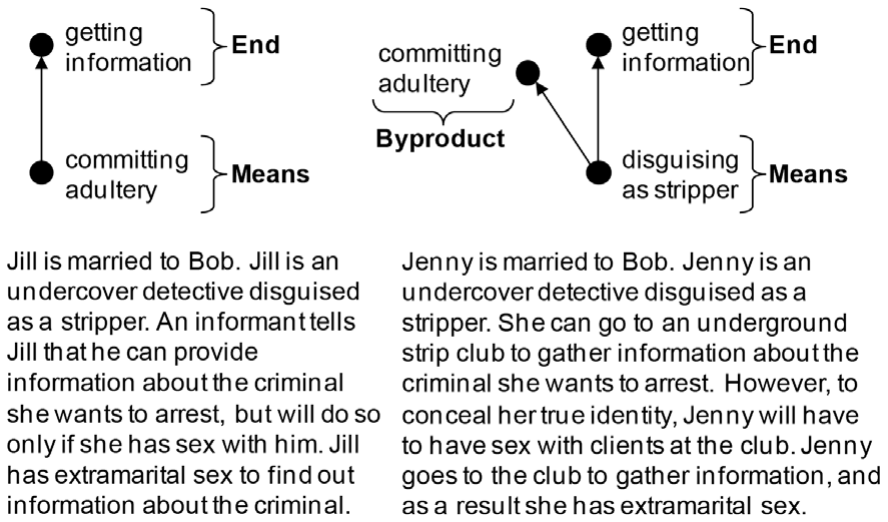
Violation as a means versus violation as a byproduct for adultery.

In Experiment 2, we examined the means-byproduct distinction across several major categories of moral violations. For each violation, we recreated the different structures embodied in the looping-track trolley problems (Fig. 1, Fig. 2). For example, in the heroin scenarios (Fig. 1), Tina takes heroin pills *in order to* relieve a mild backache (a violation as a means); Tracy wants to take aspirin for her backache but the two aspirin pills have been mixed with two heroin pills and she takes all four pills because she cannot distinguish them (a violation as a byproduct). In the means condition, like in the trolley version, the heroin was used as a means to relieve the backache – the backache would otherwise have remained – but in the byproduct condition, the aspirin was the means and heroin use was a foreseeable byproduct of taking the aspirin, like diverting the train to kill the man standing in front of the large object.

As another example, in the adultery scenarios (Fig. 2), Jill, who is married, is a detective who trades sex for information about a criminal (a violation as a means); Jenny, who is married, is a detective who disguises herself as a stripper to gather information at an underground club where strippers have sex with clients, and she has sex while at the club to keep her identity secret (a violation as a byproduct). In the means condition (like in the trolley version), the sex is traded as a *means* to get the information – she could not have gotten the information without the sex – but in the byproduct condition, disguising as a stripper was the means and having sex with clients was a foreseeable *byproduct* of the disguise. If it so happened that no clients requested sex that night, then Jenny could have gotten the information without committing adultery, because it was the stripper disguise, not the sex, that was used as a means to get the information. In contrast, it is not possible for Jill to trade sex for information without having sex, because sex was the means used to get the information.

### Method

#### Design

Experiment 2 investigated the byproduct effect using controlled pairs of violation scenarios (Methods S1). The experimental design was the same as Experiment 1 except the stimuli depicted violations committed as a means or as a byproduct. The 12 violation types were theft of $20, cheating on an exam, adultery, selling sex, buying sex, viewing child pornography, group sex, ocean pollution, euthanasia, cannibalism, heroin use, and flag-burning. The stimuli did not use the labels “means” or “byproduct.”

#### Participants, Materials, and Procedures

Participants were 95 undergraduates (62 female) enrolled in introductory psychology at the University of Pennsylvania. The mean (*SD*) age of the sample was 21.18 (2.87) years. The materials, procedures, and data analysis were the same as Experiment 1 and differed only in the scenario stimuli.

### Results

Table 3 shows mean (*SD*) wrongness judgments for means and byproduct conditions. Wrongness ratings ranged from *M* = 6.63 for flag-burning as a byproduct to *M* = 32.34 for ocean pollution as a means to an end. We conducted a paired *t*-test for each violation type. Byproducts were judged as significantly less wrong than means for six violations: adultery, cheating, euthanasia, flag-burning, pollution, and theft. There was no significant difference for the other six offenses: buying sex, cannibalism, child pornography, group sex, heroin, and selling sex.

**Table 3:** Experiment 2 wrongness judgments.

		Means		Byproduct				
Violation	*n* a	Mean	*SD*	Mean	*SD*	Difference	*t*	*p*
Adultery	73	20.53	24.74	15.33	20.49	5.21	2.10	.039
Buy Sex	81	11.56	15.32	11.14	17.23	0.42	0.31	.76
Cannibalism	82	12.40	15.24	12.46	17.50	−0.06	0.05	.96
Cheating	95	16.20	20.67	13.48	18.19	2.72	3.20	.002
Child Porn	62	12.81	18.63	10.21	15.86	2.60	1.89	.063
Euthanasia	85	11.71	17.46	7.47	15.02	4.24	4.01	<.001
Flag-burning	73	9.40	9.74	6.63	9.11	2.77	3.57	<.001
Group Sex	84	14.44	19.50	12.98	17.03	1.46	1.51	.14
Heroin	94	19.31	20.72	17.67	20.65	1.64	1.45	.15
Pollution	94	32.34	22.53	27.28	19.61	5.06	4.60	<.001
Sell Sex	83	14.73	18.29	13.89	17.00	0.84	0.72	.47
Theft	85	13.49	15.44	7.29	9.56	6.20	3.88	<.001

*Note*. All *t* tests were within-subject and two-tailed. Participants viewing neither means nor byproduct as wrong were excluded.

a Subset of total sample (*N* = 95) after excluding individuals viewing neither as wrong.

Table 4 shows mean (*SD*) punishment judgments by condition. Prison time assignments ranged from *M* = 0.13 months for euthanasia as a byproduct to *M* = 29.06 months for ocean pollution as a means. Paired *t*-tests showed that byproducts were assigned significantly less prison time than means for two offenses, cheating and theft. No significant differences were observed for the remaining ten violations.

**Table 4:** Experiment 2 punishment judgments (months in prison).

		Means		Byproduct				
Violation	*n* a	Mean	*SD*	Mean	*SD*	Difference	*t*	*p*
Adultery	73	21.62	66.75	10.83	34.92	10.79	1.53	.13
Buy Sex	81	1.80	10.34	0.60	2.99	1.20	1.20	.23
Cannibalism	82	4.49	18.44	5.16	25.76	−0.66	0.34	.74
Cheating	95	2.04	5.23	1.22	3.44	0.82	2.79	.006
Child Porn	62	5.70	22.23	2.34	13.03	3.36	1.17	.25
Euthanasia	85	0.53	2.91	0.13	1.09	0.40	1.17	.24
Flag-burning	73	2.22	14.27	1.77	14.04	0.45	1.35	.18
Group Sex	84	1.17	5.94	0.88	4.36	0.29	0.58	.57
Heroin	94	0.65	3.94	0.43	1.80	0.22	0.49	.63
Pollution	94	29.06	57.88	19.66	32.12	9.40	1.85	.067
Sell Sex	83	1.21	4.96	1.34	4.90	−0.12	0.27	.79
Theft	85	2.50	6.30	0.90	2.87	1.60	2.90	.005

*Note*. All *t* tests were within-subject and two-tailed. Participants viewing neither means nor byproduct as wrong were excluded.

a Subset of total sample (*N* = 95) after excluding individuals viewing neither as wrong.

#### Additional Analyses

Similar to Experiment 1, we observed order effects on wrongness judgments. When byproducts were judged first, there were significant byproduct effects for cheating, euthanasia, flag-burning, pollution, and theft; adultery and child porn were marginally significant. When byproducts were judged second, there were significant effects for cheating, euthanasia, flag-burning, group sex, heroin, and pollution; theft was marginally significant. Across both orders, there were no scenarios in which participants judged byproducts as worse than means.

As in Experiment 1, we tested whether the byproduct effect differed for sex-related offenses (adultery, buy sex, child porn, group sex, sell sex) compared to the other offenses by conducting a 2 (byproduct or means)×2 (sex-related or not) repeated ANOVA for wrongness judgments and we found that the byproduct by sex-related interaction was not significant, *F*(1, 989) = 2.57, *p* = .11. Paired *t*-tests showed that in aggregate the means-byproduct difference was significant for both sex-related offenses (*M* = 2.01), *t*(382) = 2.94, *p*<.01, and other offenses (*M* = 3.24), *t*(607) = 7.42, *p*<.001. For punishment judgments, this interaction was also not significant, *F*(1, 989) = 0.43, *p* = .51. Paired *t*-tests showed that in aggregate the means-byproduct difference in punishment was significant for both sex-related offenses (*M* = 2.89), *t*(382) = 1.99, *p*<.05, and other offenses (*M* = 1.86), *t*(607) = 2.20, *p*<.05.

Last, we explored whether the participant's sex influenced omission effects by using a 2 (sex)×2 (byproduct or means) repeated ANOVA for wrongness judgments. We did not find a sex by byproduct interaction, *F*(1, 989) = 0.10, *p* = .75. Similarly, we did not observe a sex by byproduct interaction for punishment decisions, *F*(1, 989) = 2.45, *p* = .12.

### Discussion

The results of Experiment 2 show that the byproduct effect is not limited to violent offenses but rather can occur across a range of moral offenses. Importantly, however, the byproduct effect did not occur in all of the moral domains we investigated. There are several potential interpretations of this observation. The byproduct effect could be specific to particular moral domains rather than spanning moral domains. Or, the byproduct effect could be sensitive to the details of a given scenario and might vary across scenarios within moral domains. Alternatively, our experimental stimuli might have failed to effectively manipulate participants' perceptions of whether the effect occurred as a means or byproduct. Experiment 3 further investigates these possibilities by using a larger sample and adding a forced-choice measure in which subjects decided which was morally worse, a violation as a means or as a byproduct.

## Experiment 3

### Method

#### Design

Experiment 3 examined the byproduct effect in a larger sample of participants recruited online. The experimental design was the same as Experiment 2, except that we added a forced-choice section in which participants answered which behavior was morally worse, the byproduct violation or the means violation. Previous work suggests that whereas there is substantial variability in cardinal measures of wrongness, there is substantial agreement about the ordinal rankings of wrongness for different moral violations [27], [28]. Therefore, a forced choice about which is more wrong is potentially appealing because it could reduce the noise associated with cardinal measures.

#### Participants, Materials, and Procedures

We recruited 183 individuals (109 female) to participate in an online study for a small payment. Participants ranged in age from 18 to 73 years. The mean (*SD*) age of the sample was 32.35 (11.03) years. The materials, procedures, and data analysis were the same as Experiment 2 and differed only in the addition of the forced-choice section.

### Results

Table 5 shows mean (*SD*) wrongness judgments for means and byproduct conditions. Wrongness ratings ranged from *M* = 9.15 for cheating as a byproduct to *M* = 50.98 for ocean pollution as a means to an end. We conducted paired *t*-tests to examine the experimental hypotheses. Byproducts were judged as significantly less wrong than means for eight violations: adultery, cheating, child pornography, euthanasia, flag-burning, heroin use, pollution, and theft. There were no significant differences observed for buying sex, cannibalism, group sex, and selling sex.

**Table 5:** Experiment 3 wrongness judgments.

		Means		Byproduct				
Violation	*n* a	Mean	*SD*	Mean	*SD*	Difference	*t*	*p*
Adultery	186	28.81	29.45	25.84	27.53	2.97	2.11	.036
Buy Sex	142	20.65	23.86	20.84	25.18	−0.19	0.14	.89
Cannibalism	154	30.19	29.76	32.01	30.54	−1.82	1.37	.17
Cheating	140	15.52	23.19	9.15	16.81	6.37	3.66	<.001
Child Porn	107	31.86	30.57	23.23	30.50	8.63	3.16	.002
Euthanasia	114	36.65	31.29	32.26	32.38	4.39	2.02	.046
Flag-burning	118	28.36	34.24	20.69	29.51	7.66	4.18	<.001
Group Sex	137	22.52	26.64	21.49	25.32	1.03	0.73	.46
Heroin	153	21.27	24.62	15.09	22.90	6.18	4.29	<.001
Pollution	197	50.98	30.83	44.42	30.93	6.56	5.86	<.001
Sell Sex	153	25.99	27.57	23.87	25.64	2.12	1.32	.19
Theft	197	28.36	30.18	23.99	28.06	4.37	3.43	<.001

*Note*. All *t* tests were within-subject and two-tailed. Participants viewing neither means nor byproduct as wrong were excluded.

a Subset of total sample (*N* = 197) after excluding individuals viewing neither as wrong.

Table 6 shows mean (*SD*) punishment judgments by condition. Prison time assignments ranged from *M* = 0.70 months for cheating as a means to *M* = 91.20 months for ocean pollution as a means. Paired *t*-tests showed that byproducts were assigned significantly less prison time than means for euthanasia, heroin use, pollution, and theft. No significant differences were observed for the remaining violations.

**Table 6:** Experiment 3 punishment judgments (months in prison).

		Means		Byproduct				
Violation	*n* a	Mean	*SD*	Mean	*SD*	Difference	*t*	*p*
Adultery	185	6.70	22.76	5.86	22.62	0.85	1.42	.16
Buy Sex	142	5.70	21.43	5.69	22.30	0.02	0.02	.98
Cannibalism	154	26.01	76.16	29.15	85.74	−3.14	0.72	.47
Cheating	140	0.70	1.97	1.32	7.44	−0.62	1.08	.28
Child Porn	106	18.88	47.63	10.81	25.80	8.07	1.84	.069
Euthanasia	114	93.61	159.26	62.14	124.04	31.48	3.28	.001
Flag-burning	118	15.75	64.29	9.82	32.38	5.93	1.36	.18
Group Sex	137	2.00	9.78	1.98	9.89	0.02	0.09	.93
Heroin	153	10.10	41.83	4.79	29.56	5.31	2.17	.032
Pollution	197	91.20	124.36	77.66	124.14	13.54	3.86	<.001
Sell Sex	153	12.64	59.79	8.29	30.54	4.34	1.56	.12
Theft	197	6.03	11.79	3.66	8.31	2.37	3.96	<.001

*Note*. All *t* tests were within-subject and two-tailed. Participants viewing neither means nor byproduct as wrong were excluded.

a Subset of total sample (*N* = 197) after excluding individuals viewing neither as wrong.

Turning to the forced-choice items, we observed a consistent byproduct effect for all twelve offenses. A greater proportion of participants judged means as morally worse than byproduct for adultery (71%, *p*<.001, binomial test), buying sex (80%, *p*<.001), cannibalism (63%, *p*<.001), cheating (87%, *p*<.001), child pornography (91%, *p*<.001), euthanasia (82%, *p*<.001), flag-burning (91%, *p*<.001), group sex (58%, *p*<.05), heroin (84%, *p*<.001), pollution (92%, *p*<.001), selling sex (68%, *p*<.001), and theft (86%, *p*<.001).

#### Additional Analyses

Similar to Experiment 2, we observed order effects on wrongness judgments. When byproducts were judged first, there were significant byproduct effects for cheating, child porn, euthanasia, flag-burning, heroin, pollution, and theft. When byproducts were judged second, there were significant effects for adultery, flag-burning, pollution, and theft; group sex and heroin were marginally significant. Across both orders, there was one case, cannibalism in the byproduct-means order, in which byproduct was judged more wrong than means.

As in previous experiments, we tested whether the byproduct effect differed for sex-related offenses (adultery, buy sex, child porn, group sex, sell sex) compared to the other offenses by conducting a 2 (byproduct or means)×2 (sex-related or not) repeated ANOVA for wrongness judgments and we found that the byproduct by sex-related interaction was significant, *F*(1, 1796) = 5.28, *p* = .022, such that the means-byproduct difference was smaller for sex-related offenses (*M* = 2.64) than for other offenses (*M* = 4.77). Paired *t*-tests showed that in aggregate the means-byproduct difference was significant for sex-related offenses, *t*(724) = 3.55, *p*<.001, and other offenses, *t*(1072) = 8.33, *p*<.001. Similarly, for punishment judgments, this interaction was significant, *F*(1, 1794) = 5.80, *p* = .016, such that the means-byproduct difference was smaller for sex-related offenses (*M* = 2.35) than for other offenses (*M* = 7.14). Paired *t*-tests showed that in aggregate the means-byproduct difference in punishment was significant for sex-related offenses, *t*(722) = 2.57, *p*<.05, and other offenses, *t*(1072) = 4.73, *p*<.001.

Last, we explored whether the participant's sex influenced omission effects by using a 2 (sex)×2 (byproduct or means) repeated ANOVA for wrongness judgments. We did not find a sex by byproduct interaction, *F*(1, 1796) = 0.60, *p* = .44. Similarly, we did not observe a sex by byproduct interaction for punishment decisions, *F*(1794) = 2.50, *p* = .11.

### Discussion

The findings of Experiment 3 further support the conclusion that the byproduct effect is not confined to violent moral offenses but can occur for a range of moral domains. The results for wrongness ratings were similar to Experiment 2 except child pornography and heroin additionally showed significant byproduct effects.

Importantly, the results from the forced-choice measure indicated byproduct effects for all twelve violations. This suggests the possibility that the byproduct effect occurs widely but is weaker and more fragile in some domains than in others. This raises questions about what might explain variation in the byproduct effect across types of violations. Future work can examine whether this variation is explained by moral domain, as was found for intentions [24], or other dimensions such as causal structure or social context, as has been found for the omission effect [5], [7].

An alternative interpretation, suggested by an anonymous reviewer, is that the observed byproduct effects were an artifact of the forced-choice task because participants were not able to indicate equal wrongness. However, if participants perceived no differences in moral wrongness among byproduct and means scenarios, then their responses would be expected to be at chance levels (50%). Hence, this interpretation does not explain why responses differed from chance in the predicted direction. Nonetheless, we recognize that the absence of an equivalence option is a possible methodological limitation of this study.

## General Discussion

Table 7 summarizes the main results across experiments. In Experiment 1, we found that the omission effect occurred in all of the moral domains that we investigated. That is, moral violations, across content domains, were viewed as less wrong and deserving of less punishment when they were the result of omission rather than commission. In Experiments 2 and 3, we found that the byproduct effect is not specific to violent offenses but occurred across a range of moral domains. When participants made cardinal judgments, indicating magnitudes of wrongness, we observed byproduct effects for roughly half of the experimental violations. When participants were asked to choose which was morally worse in a forced-choice question, byproducts were judged less wrong than means across all of the violations that we investigated. The evidence reported here answers a key question by indicating that omission and byproduct effects are not confined to violent offenses but are observed across many moral domains.

**Table 7:** Summary of omission and byproduct effects.

	Omission	Byproduct		
Violation	Exp 1	Exp 2	Exp 3	Forced-choice
Adultery	-	Y	Y	Y
Buy Sex	Y	N	N	Y
Cannibalism	Y	N	N	Y
Cheating	Y	Y	Y	Y
Child Porn	Y	Y^a^	Y	Y
Euthanasia	Y	Y	Y	Y
Flag-burning	Y	Y	Y	Y
Group Sex	Y	N	N	Y
Heroin	-	N	Y	Y
Injury	Y	-	-	-
Littering	Y	-	-	-
Marijuana	Y^a^	-	-	-
Pollution	-	Y	Y	Y
Sell Sex	Y	N	N	Y
Theft	Y	Y	Y	Y

*Note*. The table shows whether the omission or byproduct effect was significant (Y) or absent (N) in Experiments 1–3 wrongness judgments and the forced-choice in Experiment 3. (There were no significant effects in the opposite direction.)

a Indicates marginal significance.

The main finding that omission and byproduct effects occur in multiple moral domains supports the hypothesis that a single cognitive system computes moral judgments for a variety of behaviors. The present research included violations from many moral domains [12], including sexual behavior (child pornography, prostitution, group sex), dishonesty (cheating on an exam), group loyalty (flag-burning), and food (cannibalism). The occurrence of omission and byproduct effects for multiple domains suggests two possibilities: Either a common information-processing mechanism underlies multiple moral domains, or the same information-processing patterns evolved independently in different violation-specific mechanisms. The latter possibility seems unlikely and these findings therefore support the view that a single moral analyzer computes wrongness for many behaviors.

The present results do not, of course, undermine the more general idea that many distinct and distinguishable content domains are moralized. They do suggest, however, that once a content domain is moralized, the same mental processes are applied to morally-relevant behavior and the same pattern of moral judgment emerges as output, giving rise to omission and byproduct effects. In this sense, our experiments add to previous research describing characteristic features of moralization, such as outrage, censure, prohibition, internalization, overjustification, and enhanced social transmission [29].

Further, these experiments raise questions about why the byproduct effect emerged unevenly across different violations and only in forced-choice measures for some offenses. This observation could reflect variation in the byproduct effect across moral domains, variation within moral domains, or insufficient manipulation of the byproduct-means dimension. For instance, previous work showed that the omission effect can be attenuated or eliminated within a moral domain depending on the evidence for the transgression [5] and the relationship between perpetrator and victim [7]. The present experiments were designed to examine the prevalence of byproduct effects by maximizing the differences across violations. In contrast, research on variation needs to minimize the differences across violations to isolate the effects of moral domains.

To better understand variation in these effects, future research needs to hold the scenario stimuli as constant as possible across moral domains to observe the interaction with the moral domain itself and to eliminate confounds with other details that vary across scenarios (which might be difficult or impossible in some cases). For instance, a trolley scenario could be used to vary only whether the objects in the path of the trolley and the objects used to stop the trolley are people (harm), people's cars (property), sacred statues (divinity), national symbols (ingroup), or symbols of leadership (authority); the trolley framework, however, would be ill-suited for studying offenses like cannibalism or prostitution. In the present experiments, we carefully controlled the scenarios across the omission-commission and byproduct-means dimensions (Methods S1), allowing inferences about the presence of these effects, but we did not control scenarios across violation types, limiting possible inferences about the sources of variation across violation types.

If future research can isolate domain-based differences in magnitudes in omission and byproduct effects, then this could provide further insight into the underlying information-processing structure. One possibility is that variation in magnitudes could indicate that there are indeed different moral mechanisms that each independently show these effects, but to different degrees. Another possibility is that moral judgment is influenced by a number of variables and the relative impact of omissions and byproducts could depend on these other variables, which might tend to have different values across domains.

Future work can aim to disentangle precisely what drives omission and byproduct effects. For example, researchers have examined the extent to which the omission effect is driven by intentions or causality [3], [15], and the role of public transparency [5]. These questions are especially difficult in light of accumulating research showing top-down processing effects in which moral judgment influences perceptions of intentions [9], causality [30], harm [31], and designations of perpetrators and victims [32], [33], rather than the influence going only in the opposite and more intuitive direction. Although it might be convenient to use violent offenses to address these problems, a complementary approach using more diverse moral violations might yield useful insights.

Finally, future theoretical work can aim to understand the functional significance of omission and byproduct effects. Are these effects best understood as adaptations, byproducts, or performance failures? Theoretical contributions should take into account the observation that these effects operate across a diversity of moral domains, rather than occurring only in a smaller subset of violations such as violent offenses. This idea refocuses theoretical inquiries on potential functions that are facilitated by these features of moral condemnation: What functional advantages might explain these textured judgments across domains? An answer to this question might, in turn, shed light on the functions of morality.

## Supporting Information

Methods S1 **Scenario stimuli for Experiments 1–3.**
(PDF)
