# Supplementary material for: Omissions and Byproducts across Moral Domains
Source: PLoS One. 2012 Oct 11;7(10):e46963. doi: 10.1371/journal.pone.0046963 (PMC3469573; doi:10.1371/journal.pone.0046963)
Supplement: Methods S1 — Scenario stimuli for Experiments 1–3. (PDF) [file pone.0046963.s001.pdf]

Supporting Information

Omissions and Byproducts Across Moral Domains

Peter DeScioli, Kelly Asao, & Robert Kurzban

Supporting Methods S1

Stimuli for Experiment 1

Offense	Commission	Omission
Buy Sex	ALEX goes to a massage parlor. After the massage is over, Alex pays the masseuse an extra $20 to perform oral sex on him.	AARON goes to a massage parlor. After the massage is over, Aaron gives the masseuse a $20 tip. She begins to perform oral sex on him and he does not stop her.
Cannibalism	JOHN is at a restaurant in Fiji. He is given a small piece of meat. The moment before he takes a bite, he is told that the dish is made of human flesh. He takes a bite and swallows it.	JEREMY is at a restaurant in Fiji. He is given a small piece of meat. The moment after he takes a bite, he is told that the dish is made of human flesh. He swallows it nonetheless.
Cheating	SARAH is taking an exam and sitting next to Mark, who is very smart. Sarah asks Mark to tell her the correct answers, and he does so.	SALLY is taking and exam and sitting next to Mark, who is very smart. Mark tells Sally the answers and she does not stop him.
Child Porn	FRED is searching for porn on the internet. He clicks on a link that says “On the Playground” with pictures of small children. As the movie plays, it is clearly child pornography. Fred watches the movie.	FRANK is searching for porn on the internet. A pop-up appears and begins playing a movie called, “On the Playground” with pictures of small children. It is clearly child pornography. Frank does not close the pop-up window.
Euthanasia	Dr. SMITH is standing next to his patient, who has expressed a wish to die. Dr. Smith turns off the respirator, which is keeping the patient alive. The patient dies.	Dr. SCOTT is standing next to his patient, who has expressed a wish to die. The patient becomes unable to breathe on his own. Dr. Scott does not turn on the respirator, which would keep the patient alive. The patient dies.
Flag-Burning	BRYAN is American. He sees an American flag lying on the ground next to a trashcan, and he throws his cigarette on it, causing it to burn.	BRUCE is American. He throws his cigarette at a trashcan, but it falls on an American flag lying on the ground next to it. Bruce could put out the cigarette, but he does not, causing the flag to burn.
Group Sex	BILL is sitting in the living room. His wife, Jen, is in the bedroom. His wife’s friends arrive at the house, walk into the bedroom, and start having sex with Jen. Bill does not interact with Jen's friends. Bill enters the bedroom, has sex with Jen while she is having sex with her friends, and as a result, he participates in group sex.	BOB is having sex with his wife Ashley. His wife’s friends arrive at the house, walk into the bedroom, and start having sex with Ashley. Bob does not interact with Ashley's friends. Bob continues to have sex with Ashley while she is having sex with her friends, and as a result, he participates in group sex.
Injury	A man is on a ladder, fixing the roof of his house. JASON walks by and pushes the ladder down, causing the man to fall and break his leg.	A man is on a ladder, fixing the roof of his house. JACK walks by and the ladder begins to fall. Jack could stop the ladder, but he does not, causing the man to fall and break his leg.
Littering	CATHERINE is in a national park. She takes a piece of paper out of her purse, throws it on the ground, and continues walking.	CHRISTINE is in a national park. A piece of paper falls out of her purse, she sees this, and continues walking.

Offense	Commission	Omission
Marijuana	KELLY is at a party. In a small room, a group of people begins smoking a large amount of marijuana. Kelly sees this, enters the room, and, as a result, gets high from the smoke.	KRISTEN is at a party. In a small room, a group of people begins smoking a large amount of marijuana. Kristen is already in the room and stays, and, as a result, gets high from the smoke.
Sell Sex	EVA meets an attractive young man at a bar. He asks Eva to accompany him back to his hotel room and she agrees. They have consensual, protected sex. Afterwards, Eva asks man to leave $300 on the nightstand.	ELIZABETH meets an attractive young man at a bar. He asks Elizabeth to accompany him back to his hotel room and she agrees. They have consensual, protected sex. Afterwards, the man puts $300 on the nightstand; Elizabeth makes no objection.
Theft	REBECCA is shopping at a local grocery store. After she buys her items, she takes an extra $20 from the cash register while the cashier is busy. She walks out of the store with the money.	RACHEL is shopping at a local grocery store. After she buys her items, she sees that the cashier gave her an extra $20 but she does not say anything. She walks out of the store with the money.

Stimuli for Experiments 2 and 3

Offense	Means	Byproduct
Adultery	Jill is married to Bob. Jill is an undercover detective disguised as a stripper. An informant tells Jill that he can provide information about the criminal she wants to arrest, but will do so only if she has sex with him. Jill has extramarital sex to find out information about the criminal.	Jenny is married to Bob. Jenny is an undercover detective disguised as a stripper. She can go to an underground strip club to gather information about the criminal she wants to arrest. However, to conceal her true identity, Jenny will have to have sex with clients at the club. Jenny goes to the club to gather information, and as a result she has extramarital sex.
Buy Sex	Greg is an undercover cop. He wants to arrest a drug dealer who is with a prostitute at a brothel. Greg acts like a client and pays for sex in order to get inside the brothel to find the drug dealer. Greg buys sex from a prostitute in order to arrest the drug dealer.	George is an undercover cop. He wants to arrest a drug dealer who is with a prostitute at a brothel. Greg acts like a client of the brothel in order to get inside to find the drug dealer. Once inside, he calls for police backup but they take too long to arrive. To avoid blowing his cover, George buys sex from a prostitute.
Cannibalism	Annie is traveling through a remote jungle when she gets a mild headache. The locals tell her that eating a traditional soup made from human brains will relieve her migraine. Annie eats human brain in order to stop the headache.	Ashley is traveling through a remote jungle when she gets a mild headache. The locals tell her that eating a traditional soup made with a special plant will relieve her migraine. She is told that the plant is cooked with human brains to add to the flavor. Ashley eats the soup to stop the headache, and as a result she eats human brain.
Cheating	Amy must pass an exit exam with 70% or higher to graduate. She has had difficulty with the material and is unsure whether she’ll pass. During the exam, the proctor loudly discusses the correct answers for many questions. Amy writes down the proctor’s answers to make sure she passes the test.	Alice must pass an exit exam with 70% or higher to graduate. She understands the material and is sure she’ll pass. During the exam, the proctor loudly discusses the correct answers for many questions. Alice is unable to ignore the proctor’s answers, but she knows she won’t be able to reschedule the exam, so she stays and ends up writing down the proctor’s answers.

Offense	Means	Byproduct
Child Porn	Nancy is a reporter writing an article about child pornography. To learn about this, she goes to a child pornography website to watch videos of child pornography. Nancy views child pornography videos to help write her article.	Natalie is a reporter writing an article on child pornography. To learn about this, she goes to a child pornography website to read the discussion boards. The website plays videos of child pornography on the page with the discussion boards. Natalie reads the discussion boards to help write her article, and as a result she views child pornography videos.
Euthanasia	A terminally ill man is in pain. He asks his doctor to stop the pain, even if this requires his death. Dr. Jones stops the pain by giving the man a lethal injection to end his life. The man dies an hour later.	A terminally ill man is in pain. He asks his doctor to stop the pain, even if this requires his death. Dr. Jackson stops the pain by giving the man an injection of anesthesia, even though Dr. Jackson knows the side effects will end the man's life. The man dies an hour later.
Flag-burning	Timothy is talking with friends on his porch when he begins to feel cold. He looks around for material to start a fire and finds an American flag. Timothy burns the American flag to start a fire that will keep him warm.	Tommy is talking with friends on his porch when he begins to feel cold. He looks around for material to start a fire and finds an old sheet. When he pulls the sheet out, he sees that it is stuck to an American flag. Tommy lights the old sheet to start a fire that will keep him warm, and as a result he burns the American flag.
Group Sex	Paul wants to join a fraternity. To join the fraternity, participants must perform an initiation ritual that involves group sex. Paul does not want to participate in group sex, but he decides to have group sex in order to join the fraternity.	Philip wants to join a fraternity. To join the fraternity, participants must perform an initiation ritual at an exclusive party. At the party, everyone must participate in group sex or they will be kicked out. Philip does not want to participate in group sex, but he decides to have group sex so he can remain at the party to complete the initiation ritual.
Heroin	Tina has a mild backache. Heroin relieves pain. She takes two heroin pills to stop the pain.	Tracy has a mild backache. Two aspirin painkillers have been mixed with two heroin pills. She wants to take the aspirin, but she can't tell the difference. She decides to take all four pills to stop the pain, and as a result she takes two heroin pills.
Pollution	Shawn runs a toxic waste company. He can increase profits by dumping half of the waste into the ocean, rather than properly disposing of it. Shawn decides to dump toxic waste into the ocean in order to increase profits.	Steve runs a toxic waste company. He can increase profits by shipping waste across the ocean on barges. However, the barges are old and leaky so they will spill about half of the waste into the ocean. Steve decides to use the barges in order to increase profits, and as a result toxic waste is dumped into the ocean.
Sell Sex	Eve is a spy disguised as a prostitute. Eve can attend a party to get the information she needs, but the doorman, who thinks she is a prostitute, says he won't let her into the party unless she accepts him as a client. Eve exchanges sex for money in order to get into the party.	Emily is a spy disguised as a prostitute. Emily can attend a party to get the information she needs, but she will have to sell sex for money at the party to avoid blowing her cover. Emily attends the party and gets the information she needs, but in the process she sells sex for money.
Theft	James is grocery shopping. At the register, he realizes that he is $20 short of the bill. However, James wants all of the groceries, so he underpays by $20 and leaves with the groceries. James took groceries from the store without paying because he wanted them.	Jake is grocery shopping. At the register, he realizes that he is $20 short of the bill. However, Jake is late for an appointment, so he underpays by $20 and leaves with the groceries. Jake left so he wouldn't be late for an appointment, and as a result he took groceries from the store without paying.
